# The differential diagnostic value of dual-phase ^18^F-DCFPyL PET/CT in prostate carcinoma

**DOI:** 10.1038/s41391-022-00534-5

**Published:** 2022-04-14

**Authors:** Aijuan Tian, Runlong Lin, Jing Yu, Fan Zhang, Qiang Zheng, Xin Yuan, Zhanhua Sun, Zhaoyan Zhong

**Affiliations:** Department of Nuclear Medicine, The Second Hospital of Dalian Medical University, Dalian, People’s Republic of China

**Keywords:** Cancer screening, Cancer screening

## Abstract

**Objective:**

Binding of ^18^F-DCFPyL at prostate cancer (PC) cells increases over time. The dual-phase protocol may be helpful in separating benign lesions from malignant ones associated with prostate cancer. The purpose of this study was to retrospectively analyze the incremental diagnostic value of ^18^F-DCFPyL dual-time imaging in patients with prostate cancer.

**Method:**

114 prostate-related malignant lesions and 43 benign lesions in 38 patients with prostate cancer were retrospectively analyzed. Maximum standardized uptake value (SUVmax) for benign and prostate-related malignant lesions were calculated at min 60 and min 120 of PET/CT imaging. In order to calculate SUV ratio, the SUVmax of left gluteus maximus was measured at the same time. The difference of SUVmax metrics and SUV ratio between malignant and benign lesions was statistically analyzed, the cut-off value of ROC curve was calculated, and the diagnostic efficacy of SUVmax index and SUV ratio at two time points was compared.

**Results:**

SUVmax metrics and SUV ratio of early and delayed imaging of PC-related malignant lesions were significantly higher than those of benign lesions (*p* < 0.05). In terms of individual indicators, the highest accuracy and sensitivity was in the delayed SUV ratio (89.2% and 94.7%), the best specificity was in the early SUVmax (93.0%). When the individual and combined indicators were compared together, the SUV ratio in the delay period still showed the best diagnostic sensitivity and accuracy, and the best specificity were SUVmax early and ▵SUVmax, SUVmax early and RI.

**Conclusions:**

Uptake of ^18^F-DCFPyL increased over time in prostate-associated malignant lesions compared with benign tissue. For single-phase imaging, 2-hour (delayed) imaging has better diagnostic performance. However, the dual-phase imaging (1 and 2 h) are helpful in the differential diagnosis of prostate-associated malignant lesions and benign lesions.

## Introduction

Prostate cancer (PC) is considered to be the most common malignancy and the second-highest mortality rate among males [1]. Prostate-specific membrane antigen (PSMA) is a transmembrane protein found primarily in all prostatic tissues. Increased PSMA expression is seen in a variety of malignancies, most notably prostate cancer. Almost all prostate adenocarcinomas exhibit PSMA expression in most primary and metastatic lesions. ^68^Ga or ^18^F labeled PSMA has been used as a radiotracer for PC PET imaging, playing an important role in staging, diagnosing biochemical recurrence, guiding radiotherapy and surgery [2]. ^18^F-DCFPyL (2-(3-{1-carboxy-5-[(6-18F-fluoro-pyridine-3-carbonyl)-amino]-pentyl}-ureido)-pentanedioic acid) is a radioactive tracer based on the glutamate-ureido-lysine motif. ^18^F has a longer half-life of 110 min than ^68^Ga. It has been successfully used to detect PSMA expressing of prostate cancer lesions. However, PSMA expression lacks specificity in many normal tissues (eg., salivary glands, duodenal mucosa, subset of proximal renal tubular cells, and subpopulation of neuroendocrine cells) in the colonic crypts and neoplastic lesions (eg. subtypes of transitional cell carcinoma, renal cell carcinoma, colon carcinoma, and peritumoral and endotumoral endothelial cell of neovasculature). In our clinical work, we found that many benign lesions can also uptake ^18^F-DCFPyL, which has adverse effects on the imaging and diagnosis of prostate cancer. Correctly distinguishing whether it is the uptake of prostate cancer-related malignant lesions or the uptake of benign lesions is very important and a meaningful challenge for diagnosis, staging and treatment of the disease. In this study, we performed a retrospective analysis of ^18^F-DCFPyL uptake in a number of known benign and malignant lesions. It was hoped to provide theoretical and data basis for distinguishing these two lesions.

## Materials and methods

### Patients and pathology

The study was approved by the Ethics Committee of the Second Hospital of Dalian Medical University (2021-XJS-24), and all patients had informed consent. Patients who were not obtained in the scheduled time‐frame, had incomplete clinical data, or whose lesions did not intake the radiotracer due to heterogeneity were excluded. Finally, 38 patients with pathological diagnosis of prostate cancer underwent dual-phase ^18^F-DCFPyL PET/CT in the Department of Nuclear Medicine of the Second Hospital of Dalian Medical University from 2018 to 2019 were included in the study. The characteristics of patients investigated in this study was showed in Table 1. There were 19/38 patients with a biochemical relapse of PC, the median Gleason score was 8 (range 7–9), and the median PSA value was 1.06 μg/L (range 0.02–19.36 μg/L) at PET/CT examination. The 12/38 patients were for primary staging, the median Gleason score was 9 (range 7–10), and the median PSA value was 67.20 μg/L(range 4.50–272.40 μg/L). The purpose of another 7/38 patients was efficacy evaluation of androgen deprivation therapy, the median Gleason score was 9 (range 7–9), and the median PSA value was 40.04 μg/L(range 0.20–112.90 μg/L).

**Table 1:** Characteristics of all patients investigated in this study (*n* = 38).

Characteristic	Age y)	Gleason score	PSA at PET (μg/L)
Range	54–82	7–10	0.02–272.40
Mean	71.4	8.2	29.73
SD	6.4	0.9	55.63

### Synthesis of ^18^F-DCFPyL

^18^F-DCFPyL was synthesized by an on-site cyclotron and radiochemistry facility at the Nuclear Medical Department of the Second Hospital of Dalian Medical University. The radiosynthesis of ^18^F-DCFPyL was performed on a domestic PET-MF-2V-IT-I synthesis module. ^18^F Fluoride ion was produced by the ^18^O(p, n)^18^F nuclear reaction using a Sumitomo HM-10 cyclotron. The precursor of DCFPyL was 5-(((S)-6- (tert-butoxy) -5-(3-((S)-1,5-di-tert -butoxy -1,5-dioxopentan -2-yl) ureido)-6-oxohexyl) carbamoyl) -N,N,N -trimethylpyridin -2-aminium trifluoromethane-sulfonate. Radiochemical yield of ^18^F-DCFPyL was (7.90 ± 0.73)% (decay not corrected), and the radiochemical purity was >95% in all cases.

### Imaging Protocol

No fasting was required and no diuretics were administered prior to imaging. The administered activity was scaled according to the patient’s body weight (range, 3.70–5.55 MBq/Kg). PET images were acquired from head to mid thigh on a PET/CT (Ingenuity TF, Philips) at 60 and 120 (+/− 5 min) min post-intravenous injection (p.i.). All images acquired with the PET/CT system were evaluated using the scatter-corrected (SC) and attenuation-corrected (AC) PET images, as is common practice. CT scan for localization and attenuation correction 120 kV, automatic milliamperage selection (range, 25–150 mA). Immediately after the CT scan, PET data was acquired at 3 min/bed. Repeat these operations at min 120. Attenuation correction was performed using the low-dose non-enhanced CT data.

We performed the reconstructions of data set with slice thickness of 4 mm. The clinical standard reconstruction consisting of the iterative proprietary BLOB-OS-TF algorithm provided by the PET/CT scanner manufacturer, including reconstructed time-of-flight information.

### Image analysis and quantification

Two experienced nuclear medicine physicians independently interpreted the PET/CT images. Excellent Inter-rater agreement was noted between two physicians in visual analysis of dual-phase images. In case of disagreement between the two, a consensus would be achieved after detailed discussion. Reconstructed images were displayed on a dedicated workstation equipped with Philips InteliSpace Protal.

Reference standard for benign and malignant lesions was determined by histopathology and changes in imaging, clinical findings, and biochemical parameters at 6 months (plus or minus 30 days) [3]. Cases were considered prostate cancer-related malignant lesions if one of the following hard criteria was met: histopathology showed prostate adenocarcinoma, or the bone lesion became sclerosing or bursting on follow-up imaging. Cases were also considered malignant if at least three soft criteria were met. These included (1) typical appearance of multi-focal metastatic disease; (2) a metastatic lesion on an imaging modality; (3) increase in size or number of lesions from one imaging exam to the next; (4) decrease in size or number of lesions from one imaging exam to the next, following appropriate treatment; (5) lesion associated with clinical symptoms suggesting malignancy; (6) patient received localised treatment for imaging finding; (7) increase in PSA in keeping with clinical scenario of progression, or decrease in response to treatment; and (8) unequivocal persistence of positive finding on repeating imaging at 6 months in patients with a PSA concentration of more than 0.2 μg/L at least 3 weeks following prostatectomy. If the above conditions were not met, it was determined to be benign.

The radiotracer biodistribution was simiquantified by the maximum of standardlized uptak value (SUVmax), SUV ratio, ▵SUVmax, retention index (RI) and Ratio (SUVratio delay/early) for lesions visible on both time points.

Standardlized uptak value (SUV) was calculated using lean body mass as follows:

SUV = radioactivity in regions of interest (ROI) (Bq/ml) × lean body mass (kg)/injected radioactivity (Bq). To minimize partial-volume effects, the SUVmax within ROIs was used.

SUVratio = SUVmax of focus/SUVmax of left gluteus maximus

▵SUVmax = SUVmax delay-SUVmax early

RI = (SUVmax delay–SUVmax early/SUVmax early) × 100%

Ratio (SUVratio delay/early) = SUVratio delay/SUVratio early

The visible ^18^F-DCFPyL uptake focus were analyzed qualitatively (malignant or benign), quantitatively (SUVmax), and location (prostate, lymph node, bone, soft tissue and others) were also included in the analysis.

### Statistical analysis

SPSS version 22.0 was used for statistical analysis. The semiquantitative values were expressed as means with Standard Deviation. Differences of prostate-related malignant lesions and benign lesions in SUVmax between 60 min p.i. (early) and 120 min p.i. (delay) scan, ▵SUVmax, RI and SUV ratios were evaluated using two-sample t-test for independent samples. Levene’s test for equality of variance showed the variance similar between the groups. *P*-value less than 0.05 was regarded as statistical significance. The Receiver Operating Characteristic (ROC) curve was obtained by plotting sensitivity against (1-specificity) for SUVmax metrics and SUV ratios. Youden index, the difference between the sensitivity (true positive rate) and 1-specificity (false positive rate) were used for identification of the optimal cut-off threshold for differential diagnosis of benign and malignant uptake of ^18^F-DCFPyL. Diagnostic accuracy was assessed by the area under the ROC curve (AUC). The sensitivity, specificity, positive predictive value, negative predictive value and total accuracy rate were calculated by fourfold tables.

## Result

### The SUVmax metrics and SUV ratios in malignant lesions were significantly higher than the benign lesions

**Fig. 1:**
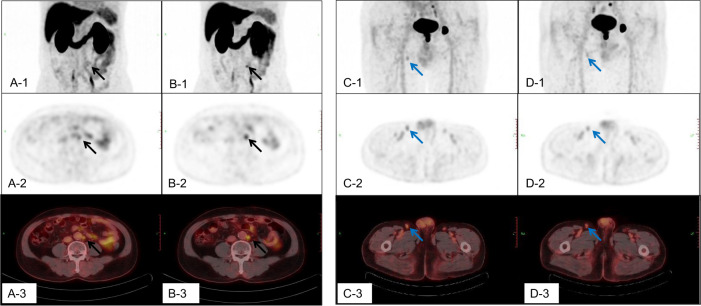
The ^18^F-DCFPyL uptake of malignant and benign lymph nodes in dual-phase PET/CT imaging. The A,B,C, D-1 were the maximum intensity projection (MIP) images; The A,B,C, D-2 were the cross sections of PET; and the A,B,C, D-3 were the cross sections of fusion images. The A, C-1, 2, 3 were for the early phase, and the B, D-1, 2, 3 were for delay phase. The A and B were the dual-phase ^18^F-DCFPyL PET/CT scan for a retroperitoneal lymph node metastasis. The short diameter of this lymph node was about 7 mm, SUVmax early was 3.0, SUVmax delay was 3.8, △SUVmax was 0.8, RI was 26.7%, SUV ratio early was 5.0, SUV ratio delay was 12.7, and ratio(SUV ratio delay/early) was 2.54. The C and D were the dual-phase ^18^F-DCFPyL PET/CT scan for a reactive hyperplastic lymph node. The short diameter of this lymph node was about 7 mm, SUVmax early was 2.1, SUVmax delay was 1.7, △SUVmax was -0.4, RI was -19.0%, SUV ratio early was 3.5, SUV ratio delay was 3.4, and ratio (SUV ratio delay/early) was 0.97.

Thirty-eight patients(mean age 71 years, range: 54–82 years) underwent dual-phase ^18^F-DCFPyL PET/CT. A total of 157 lesions uptaking ^18^F-DCFPyL were included in the analysis. Based on the pathological results, clinical follow-up included contrast-enhanced CT, magnetic resonance imaging (MRI), bone scintigraphy (BS), and repeated ^18^F-DCFPyL PET/CT to confirm response to treatment of the initial suspicious lesion, which was classified as prostate-related malignant lesions (*n* = 114) and benign lesions (*n* = 43). The SUVmax metrics and SUV ratios of malignant lesions were significantly higher than those of benign lesions (*p* < 0.05). In the comparison among the different distributions of prostate-related malignant lesions, only the RI value of metastatic lesions was significantly higher than that of primary lesions (*p* = 0.001), and there was no significant difference in SUVmax early, delay phase, ▵SUVmax, and SUV ratios (*p* > 0.05). For benign lesions, the Ratio(SUV ratio delay/early) of lymph nodes and bones was higher than that of other locations (*p* < 0.05), and there was no significant difference in other indicators among different locations of benign lesions (Tables 2–4 and Fig. 1).

**Table 2:** SUVmax metrics and SUV ratios in malignant and benign lesions (*n* = 157).

Variable	Group		*P* value
	Malignant (*n* = 114)	Benign (*n* = 43)	
SUVmax early	10.9 ± 12.5	2.7 ± 3.7	0.000^a^
SUVmax delay	14.6 ± 16.7	2.9 ± 4.0	0.000^a^
△SUVmax	3.7 ± 5.1	0.2 ± 1.2	0.000^a^
RI(%)	34.0 ± 39.3	9.9 ± 64.3	0.005^a^
SUV ratio early	22.5 ± 26.3	5.4 ± 5.3	0.000^a^
SUV ratio delay	33.4 ± 36.1	7.2 ± 9.7	0.000^a^
Ratio(SUV ratio delay/early)	1.6 ± 0.7	0.9 ± 0.5	0.000^a^

Values presented as mean ± SD or number.

^a^stands for significant difference between the two groups (*p* < 0.05).

**Table 3:** Distribution of malignant lesions, SUVmax metrics and SUV ratios (n = 114).

Lesions	Number	SUVmax early	SUVmax delay	△SUVmax	RI (%)	SUV ratio early	SUV ratio delay	Ratio (SUV ratio delay/early)
Primary lesion	16	10.3 ± 5.4	13.3 ± 7.5	3.0 ± 2.6	25.7 ± 20.9	21.4 ± 10.8	34.6 ± 19.2	1.6 ± 0.4
Metastatic lesions	98	11.0 ± 13.3	14.9 ± 17.8	3.9 ± 4.1	66.4 ± 105.7	22.7 ± 28.0	33.1 ± 38.2	1.6 ± 0.7
Local invasion	9	9.5 ± 5.6	14.2 ± 9.2	4.7 ± 4.4	45.9 ± 41.4	16.0 ± 8.6	26.6 ± 15.9	1.7 ± 0.5
Bone	47	12.3 ± 13.4	16.3 ± 17.4	4.2 ± 4.4	59.2 ± 69.3	22.6 ± 25.0	33.2 ± 33.8	1.6 ± 0.8
Lymph node	38	10.5 ± 15.0	14.2 ± 20.4	3.74 ± 4.0	69.7 ± 140.8	20.8 ± 22.9	30.7 ± 33.2	1.6 ± 0.6
other	4	4.0 ± 2.8	4.8 ± 2.9	3.5 ± 2.0	164.8 ± 154.0	11.7 ± 14.2	14.4 ± 13.2	1.7 ± 0.6
*P* value ^*a*^		0.823	0.725	0.416	0.001*	0.859	0.880	0.763
*P* value ^*b*^		0.811	0.791	0.692	0.145	0.319	0.511	0.988

Values presented as mean ± SD or number.

^a^Comparison of primary and metastatic lesions, independent sample T test was used analyze.

^b^Comarison of malignant lesions in various parts, one-way ANOVA; * stands for significant difference between the groups (*p* < 0.05).

**Table 4:** Distribution of Benign lesions SUVmax metrics and SUV ratios (*n* = 43).

Lesions	Number	SUVmax early	SUVmax delay	△SUVmax	RI (%)	SUV ratio early	SUV ratio delay	Ratio SUV ratio early/delay)
Lymph node	13	3.5 ± 6.6	3.2 ± 6.9	−0.4 ± 0.3	−22.9 ± 11.4	7.0 ± 8.9	7.9 ± 16.3	1.3 ± 0.5
Bone	2	2.2 ± 0.6	1.5 ± 0.1	−0.7 ± 0.8	−26.0 ± 28.5	5.2 ± 2.9	4.6 ± 1.3	1.3 ± 1.0
Inflammatory and postoperative change	7	1.7 ± 0.4	1.7 ± 0.9	−0.1 ± 0.9	−1.2 ± 55.3	3.0 ± 0.4	3.3 ± 2.0	0.8 ± 0.2
Nerve	2	1.6 ± 0.2	2.0 ± 0.4	0.4 ± 0.6	28.6 ± 40.4	2.9 ± 0.4	5.4 ± 1.0	0.6 ± 0.2
Ejaculatory duct	2	2.7 ± 0.0	3.2 ± 0.1	0.5 ± 0.1	16.7 ± 2.6	7.5 ± 0.6	12.8 ± 7.1	0.7 ± 0.4
Benign tumor	6	2.0 ± 1.1	2.1 ± 0.9	0.1 ± 0.8	18.8 ± 54.0	3.3 ± 1.3	4.1 ± 1.4	0.8 ± 0.4
Other	11	3.0 ± 1.6	4.1 ± 2.4	1.1 ± 1.9	52.7 ± 98.8	6.5 ± 2.8	10.9 ± 5.2	0.7 ± 0.3
*P* value^*a*^		0.956	0.900	0.093	0.142	0.602	0.722	0.015^a^

Values presented as mean ± SD or number; ^a^ Comarison of malignant lesions in various parts, one-way ANOVA.

^a^stands for significant difference between the groups (*p* < 0.05).

### Evaluation of individual indicators, the SUVmax early and SUV ratio delay showed better diagnostic performance

**Fig. 2:**
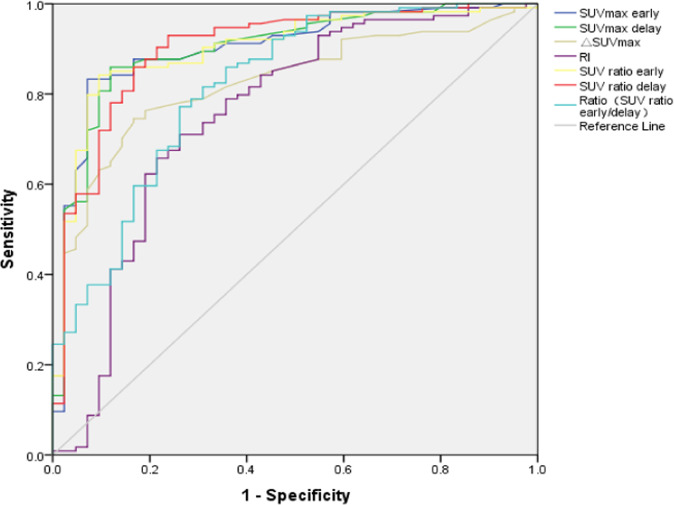
The ROC curve of SUVmax metrics for Differential Diagnosis Malignant and Benign Lesions. The area under curve (AUC) for SUVmax early was 0.904, SUVmax delay was 0.903, △SUVmax was 0.829, RI was 0.759, SUV ratio early was 0.903, SUV ratio delay was 0.902, and Ratio(SUV ratio delay/early) was 0.818.

The area under the receiver operating characteristic (ROC) curve was calculated for SUVmax metrics and SUV ratios (Fig. 2). The cut-off value of each metrics was calculated according to the ROC curve, and the sensitivity (Sn), specificity (Sp), positive predictive value (PPV), negative predictive value (NPV), and accuracy were also calculated (Table 5). The cut-off value of 7.0 for SUV ratio delay showed the highest Sn (94.7%), NPV (84.2%), and accuracy (89.2%). The cut-off value of 3.5 for SUVmax early showed the best Sp (93.0%) and PPV (96.9%). Other independent indicators were not dominant in diagnosis.

**Table 5:** The cut-off values and diagnostic efficacy of SUVmax metrics and SUV ratio for differential diagnosis malignant and benign lesions.

	Cut-off value	Sn (%)	Sp (%)	PPV (%)	NPV (%)	Accuracy (%)	AUC
SUVmax early	3.5	83.3	93.0	96.9	67.8	86.0	0.904
SUVmax delay	3.7	86.0	86.0	94.2	69.8	86.0	0.903
△SUVmax	0.7	74.6	86.0	93.4	56.1	77.8	0.829
RI(%)	15.6	71.9	74.1	88.2	50.0	72.6	0.759
SUV ratio early	8.0	85.1	88.4	95.1	69.1	86.0	0.903
SUV ratio delay	7.0	94.7	74.4	90.8	84.2	89.2	0.902
Ratio(SUV ratio delay/early)	1.1	86.0	83.7	93.3	69.2	85.3	0.818

Values presented as mean ± SD or number (%); Sn = Sensitivity; Sp = Specificity; PPV = Positive predictive value; NPV = Negative predictive value; AUC = Area under curve.

### Evaluation the individual and combined indicators together, SUVratio delay, SUVmax early and ▵SUVmax, SUVmax early and RI showed better diagnostic efficacy in all the indicators

**Fig. 3:**
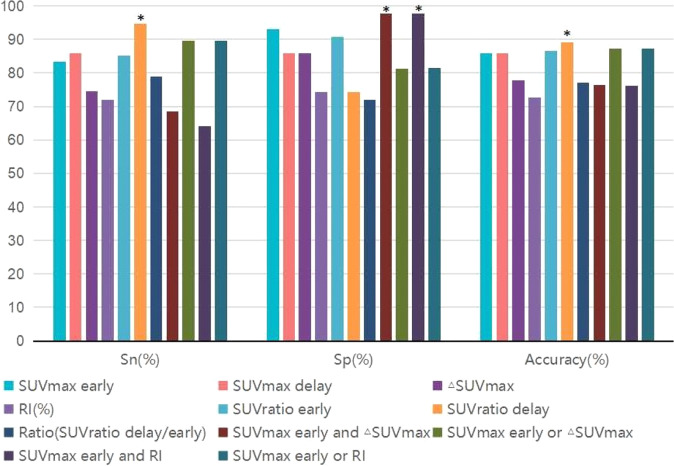
Diagnostic efficiency for individual and combined indicators SUVmax metrics in differential diagnosis of benign and malignant lesions (*n* = 157). A total of 7 individual indicators and 4 combined indicators were included in the comparison of diagnostic efficacy.meant the best indicator. For sensitivity (Sn), that was the ability to diagnose malignant lesions, SUVratio delay was the best performer (94.7%). For specificity (Sp), which was used to diagnose benign lesions, two combined indicators (SUVmax early and △SUVmax, SUVmax early and RI) performed equally well (97.7%). In terms of overall accuracy, SUVratio delay was still the best diagnostic criterion (89.2%).

Totally 4 combined SUVmax indicators were included in the analysis. SUVmax early and ▵SUVmax, SUVmax early, and RI meant that both criteria must be met at the same time to make a diagnosis. SUVmax early or ▵SUVmax, SUVmax early or RI meant a diagnosis could be made if either of the two criteria was met. In terms of combined indicators comparison, the SUVmax early or RI had the highest Sn (89.5%) and accuracy (87.2%), the SUVmax early and ▵SUVmax, SUVmax early, and RI the had the highest Sp(97.7%). But in terms of comparison of all indicators, the highest Sn and accuracy belonged to the SUV ratio delay (94.7%, 89.2%) yet, and the best specificity were still the SUVmax early and ▵SUVmax, SUVmax early and RI (Fig. 3).

## Discussion

The outcome of PC is largely determined by distant metastasis, which can occur via lymphatic and hematogenous pathways and almost any distant organs can be targeted. Bone, liver, lung and lymph nodes are the most common metastatic sites. PC can also locally invade adjacent organs such as the urinary bladder [4]. Detection of primary lesions, local invasion and metastasis is important for staging of newly diagnosed PC patients and management of patients with biochemical recurrence. Although PSMA PET has obvious advantages in the diagnosis and staging of PC, it is still a challenge to accurately determine whether radiotracer uptake is a prostate-related malignant lesion or benign lesion.

PSMA reported functions include enzymatic peptidase activity related to folate and glutamate metabolism, as well as activation of signaling pathways (e.g., Akt and MAPK) involved in cell proliferation and survival. Endothelial PSMA expression has been extensively studied and occurs in nearly all nonprostatic solid tumors associated with neovasculature [5]. Some research have shown that PSMA expression of immune cells related to an increase in regional blood flow/vascular permeability in the location of inflammation/infection. Furthermore, some inferences about macrophage folate receptors and their potential implications in PSMA ligand imaging has been suggested [6]. Although it is manifested as low to moderate uptake of radiotracer in non-prostate related bone diseases, such as osteodegenerative changes, fracture and osteomyelitis, some lesions may show intense uptake, most of them are vascular origin, such as bone and hepatic hemangioma [7, 8]. Soft tissue uptake in the surgical site is seen after prostate surgery, which may be related to reparative inflammatory processes and/or increased blood vessels, and is often misinterpreted as a local residual tumor. Some tumors of neurogenic origin also can uptake the tracers, such as meningiomas, schwannomas, and neurofibromas [9–11]. PSMA expression by nonprostatic malignant neoplasms has been confirmed by immunohistochemistry studies. Many cancers, such as lung, renal, colorectal, gastric and thyroid cancers, sarcomas, lymphomas and other tumors had been reported can uptake the PSMA tracter.

Therefore, PSMA expression can be found in several tissues and/or conditions, including normal nonprostatic epithelial cells, inflammation, infection, nonprostatic neoplastic cells and nonprostatic tumor-associate neovasculature [12–20]. Strong PSMA tracer uptake is usually observed in lacrimal, parotid and submandibular glands, small intestine, kidney, liver, spleen and bladder. Mild to moderate uptake maybe observed in nasal and esophageal mucosa, the vocal cords, gallbladder and billary tract, tracheal and proximal bronchi, mediastinal, axillary and inguinal lymph nodes, gynecomastia, and sympathetic ganglia(such as stellate, celiac, hypogastric and sacral ganalia). Some benign changes in bone, infection and inflammation (including postoperative changes) also uptake the tracer. Many normal tissues, such as hyperplastic lymph nodes and ganglia have also been reported to be able to uptake the PSMA radiotracers [21–23]. Finding imaging features of prostate-related malignant lesions and benign uptake is the key to differential diagnosis.

SUV measurement is the most common and convenient indicator for PET/CT to judge the benign and malignant lesions. Many factors may affect the SUV measurements. Biological factors include body size, post-injection uptake time, and respiratory motion, and technical factors include inter-scanner variability and image reconstruction parameters, and so on [24]. In ^18^F-FDG PET/CT imaging, dual-phase imaging is helpful to distinguish the benign and malignant lesions, can the same effect be reflected in PSMA PET imaging? Although there are studies on PSMA PET biphasic imaging, ie, 3-6 min and 1 h biphasic, only to improve the detection of locally involved pelvic disease, and 3-6 min imaging is also difficult to implement [25]. Can the differential diagnosis of lesions distributed throughout the body be resolved with a routine inspection rhythm? This study is to explore this problem.

In our study, we chose two kinds of semiquantitative indications, SUVmax values and SUV ratio. SUVmax is commonly used for semiquantitative assessments found in PET/CT and is the highest voxel value within the ROI, so it is independent of ROI definition (assuming the voxel with the highest activity concentration is included). Currently, SUVmax is most commonly used because it is less observer-dependent and more reproducible than SUVmean. For SUVmax indicators, we choose the SUVmax early, SUVmax delay, ▵SUVmax and RI as evaluation contents, which are the most commonly used indexes for PET dual-phase imaging, and routinely obtained in our diagnostic work. The SUV ratio, which also had been used in other studies to differentiate benign from malignant lesions [26, 27]. We found that all the Individual indicators of malignant lesions were significantly higher than those of benign lesions (*p* < 0.05). In our study, the most common focus of benign PSMA uptake were reactive lymph nodes and postoperative change, which are important for diagnosing local recurrence and lymph node metastasis, and are difficult to diagnose with other imaging modalities. We found that their PSMA uptake were significantly lower than that in malignant lesions (Tables 3, 4). Among malignant lesions, the RI of metastatic lesions was significantly higher than that of primary lesions (*p* < 0.05), and there were no significant difference between primary and metastatic lesions in other indicators. There were no differences in the indicators between different benign lesions. This means that the intensity of imaging tracer uptake can help differentiate benign focus from malignant lesions of PC, and RI may be helpful for differential diagnosis of primary PC focus and benign lesions such as prostatic hyperplasia, inflammation and postoperative change. Then, we calculated the cut-off value of individual indicator with ROC curve. Take the cut-off value as the diagnostic criterion, we found that among these indicators, the most sensitive was SUV ratio delay (cut-off value 7.0, 94.7%), and the most specific was SUVmax early (cut-off value 3.5, 93.0%). It seems that 120 min imaging was more conducive to the diagnosis of malignant lesions, and 60 min imaging was better in diagnosing benign lesions. While these diagnostic performance were already very good, could the combined indicators further improve the diagnostic performance? In this study, we involved 4 combined diagnostic indicators. We compared the diagnostic efficiency of individual and combined indicators. In terms of the diagnostic accuracy of malignant lesions (sensitivity), delayed imaging indicator (SUV ratio delay) still had advantage (94.7%). This meant that the increased uptake of the ^18^F-DCFPyL that was visible in delayed imaging could help the diagnosis of malignant lesions. But the combination indicators also showed their superiority. Both (SUVmax early and ▵SUVmax) and (SUVmax early and RI) showed best specificity (97.7%), they were the most effective in diagnosing benign lesions.

Then, we sorted out the these results. In this study, comparison of 1 h imaging and 2 h imaging, the 2 h PET/CT imaging was more conducive to finding malignant lesions, and the intensity and change of imaging agent intake were more conducive to the diagnosis of benign change from malignant focus of prostate gland. Many studies have confirmed that for PSMA PET imaging at 2 or 3 h post-injection may improve tumor lesion detection compared to earlier time-point imaging [28–30]. Dual-phase PSMA PET/CT imaging allowed a clear differentiation between malign and benign findings [31].

In this study, the diagnostic efficiency of 2-hour imaging was the best. In fact, both benign and malignant lesions were visible at the 1 h p.i. image in this study. However, in the case of doubts about the nature of the lesion, we performed a delayed (2 hr p.i.) imaging of the local or trunk as appropriate. In the 2 hr p.i. image, the prostate-related malignant lesions were clearer (SUVratio delay increased), while the benign lesions became relatively blurred. Jansen et al. showed that ^18^F‐DCFPyL uptake in PCa metastases rises continuously during the first 2 h after injection, while background activity decreases. Hence, the contrast between tumor and background will increase over time [32]. Wondergem et al indicated that by visual analysis, 38.5% of all patients show more lesions using images 120 min p.i. as compared to 60 min p.i. images, and in 9.2% a change in TNM‐staging was found. All lesions seen on images 60 min p.i. were also visible on images 120 min p.i..A significant better mean signal to noise ratio (SNR) was found for images acquired 120 min p.i. than 60 min p.i. [33]. Our findings support the observations of them. Our observations may help decide whether an incipient metastasis is present or not in ambiguous findings on ^18^F-DCFPyL PET/CT. Which obviously increased in prostate-related malignant tissues, but there were little or no increase or even decrease in benign tissues. It was very helpful for differentiation. Through the changes of radiotracer intake intensity of the two phase imaging, we have improved the accuracy of differential diagnosis through the simplest and most intuitive methods, provides a more accurate staging for the clinic.

As far as the patient was concerned, the increased radiation dose was only a low-dose CT of local or trunk, but the benefit was more accurate diagnosis, staging and evaluation. From the perspective of minimizing the absorbed dose to the patient, we recommend that delay imaging (e.g., 2 h p.i.) should be used only in cases of unclear lesions. The PET/CT image acquisition speed has been relatively fast at present. In terms of medical work arrangements are concerned, only 5-10 min were added to the routine PET examination process, which dose not affect the overall examination rhythm. However, by optimizing the contrast of lesions and delaying imaging information, diagnostician can make more accurate judgments, resulting in better detection rates. In general, both patients and doctors benefit. The physical half-life of ^18^F is 110 min. Previous studies on ^68^Ga (physical half-life of 68 hr) have shown that too long-term imaging time will increase imaging noise [34], and the patient stayed in nuclear medicine department for too long, so we do not recommend 3 hr imaging. Therefore, we suggested that on the basis of conventional 1 hr p.i. imaging, for suspicious lesions, a 2 hr p.i. delayed imaging was recommended to further clarify the nature of the lesions.

Adequate reproducibility of SUV can be achieved by harmonizing patient preparation as well as acquisition and reconstruction parameters as recommended by the European Association Research Ltd (EARL) accreditation program, the North American Quantitative Imaging Biomarker Alliance (QIBA), and Uniform Protocols in Clinical Trials (UPICT), those are all based on ^18^F-FDG imaging. For ^18^F-DCFPyL, a unified image acquisition and processing standard has not been established yet. Therefore, the cut-off value of SUV calculated in this study was limited to the center, the equipment, the PSMA imaging agent, the acquisition and reconstruction protocol, and so on. Other centers and equipments should calculate the corresponding cut-off value according to their own collection habits. However, the differences in SUV value changes between benign and malignant lesions shown in this study (▵SUVmax, RI and Ratio) were definitive and had differential diagnostic significance. It will provide support and inspiration for subsequent studies on the differential diagnosis of benign and malignant lesions. Now artificial intelligence(AI) technology is developing rapidly. As far as PET/CT diagnosis is concerned, the study of SUV changes is an indispensable part of AI. There were few studies on the changes in the value of ^18^F-DCFPyL PET SUV targeted by prostate cancer. A larger sample of patients should be performed and artificial intelligence will also be used in our future studies.

## Conclusion

Compared with benign tissues, the uptake of ^18^F-DCFPyL in prostate-related malignant lesions increases over time. As far as single-phase imaging is concerned, 2-hour (delayed) imaging has better diagnostic performance. However, the dual-phase imaging (1 and 2 h) imaging may be helpful for more precise evaluation of benign and prostate-related malignant lesions. SUV ratio delay, SUVmax early combined with RI and ▵SUVmax are good indicators for differential diagnosis.

## Data Availability

Some or all data, models, or code generated or used during the study are available from the corresponding author by request.

## References

[1] Siegel RL, Miller KD, Jemal A (2020). Cancer statistics, 2020. CA Cancer J Clin.

[2] Fendler WP, Eiber M, Beheshti M, Bomanjial J, Ceci F, Cho S (2017). 68Ga-PSMA PET/CT: Joint EANM and SNMMI procedure guideline for prostate cancer imaging: version 1.0. Eur J Nucl Med Mol Imaging.

[3] Hofman MS, Lawrentschuk N, Francis R, Tang C, Vala I, Thoms P (2020). Prostate-specific Membrane Antigen PET-CT in patients with high-risk Prostate Cancer Before curative-intent Surgery or Radiotherapy (proPSMA): a prospective, randomised, multi-centre study. Lancet.

[4] Bubendorf L, Schopfer A, Wagner U, Ssuter G, Moch H, Willi N (2000). Metastatic patterns of prostate cancer: Aan autopsy study of 1589 patients. Hum Pathol.

[5] O’Keefe DS, Bacich DJ, Huang SS, Heston WDW (2018). A perspective on the evolving story of PSMA biology, PSMA-based imaging, and Endoradiotherapeutic strategies. J Nucl Med.

[6] Hermann RM, Djannatian M, Czech N, Nitsche M (2016). Prostate-specific membrane antigen PET/CT: false-positive results due to sarcoidosis?. Case Rep. Oncol.

[7] Artigas C, Otte FX, Lemort M, Velthoven RV, Flamen P (2017). Vertebral hemangioma mimicking bone metastasis in 68Ga-PSMA Ligand PET/CT. Clin Nucl Med.

[8] Bhardwaj H, Stephens M, Bhatt M, Thomas PA (2016). Prostate-specific membrane antigen PET/CT findings for hepatic hemangioma. Clin Nucl Med.

[9] Courtney M, Johnston C, Nasoodi A (2021). Meningioma uptake of 68 Gallium-PSMA-11 as a pitfall on positron emission tomography/computer tomography. Acta Radio Open.

[10] Dias AH, Bouchelouche K (2018). Prostate-specific membrane antigen PET/CT incidental finding of a schwannoma. Clin Nucl Med.

[11] Gulhane B, Ramsay S, Fong W (2017). 68Ga-PSMA uptake in neurofibromas demonstrated on PET/CT in a patient with neurofibromatosis Type 1. Clin Nucl Med.

[12] Barbosa F, Queiroz MA, Nunes RF, Costa LB, Zaniboni EC, Marin J (2020). Nonprostatic diseases on PSMA PET imaging: a spectrum of benign and malignant findings. Cancer Imaging.

[13] Keidar Z, Gill R, Goshen E, Israel O, Davidson T, Morgulis M (2018). 68Ga-PSMA PET/CT in prostate cancer patients – patterns of disease, benign findings and pitfalls. Cancer Imaging.

[14] Perez PM, Flavell RR, Kelley RK, Umetsu S, Behr SC (2019). Heterogeneous uptake of 18F-FDG and 68Ga-PSMA-11 in hepatocellular carcinoma. Clin Nucl Med.

[15] Shetty D, Han L, Bui C, Mansberg R, Stevanovic A (2016). Elevated 68Ga prostate specific membrane antigen activity in metastatic non-small cell lung cancer. Clin Nucl Med.

[16] Damle NA, Bal C, Singh TP, Gupta R, Reddy S, Kumar R (2018). Anaplastic thyroid carcinoma on 68Ga-PSMA PET/CT: opening new frontiers. Eur J Nucl Med Mol Imaging.

[17] Damle NA, Tripathi M, Chakraborty PS, Sahoo MK, Bal C, Aggarwal S (2016). Unusual uptake of prostate specific Tracer 68Ga-PSMA–HBED-CC in a benign thyroid nodule. Nucl Med Mol Imaging.

[18] Ardies PJ, Gykiere P, Goethals L, Mey JD, Geeter FD, Everaert H (2017). PSMA uptake in mediastinal sarcoidosis. Clin Nucl Med.

[19] Dekker I, Leest MVD, Rijk MCV, Gerritsen WR, Arens AIJ (2018). 68Ga-PSMA uptake in angiolipoma. Clin Nucl Med.

[20] Sasikumar A, Joy A, Nanabala R, Pillai M, Hari TA (2016). 68Ga-PSMA PET/CT false-positive tracer uptake in paget disease. Clin Nucl Med.

[21] Afshar-Oromieh A, Sattler LP, Steiger K, Holland-Letz T, Haberkorn U (2018). Tracer uptake in mediastinal and paraaortal thoracic lymph nodes as a potential pitfall in image interpretation of PSMA ligand PET/CT. Eur J Nucl Med Mol Imaging.

[22] Klingenberg S, Jochumsen MR, Nielsen TF, Bouchelouche K (2019). 68Ga-PSMA uptake in anal fistula on PET/CT Scan. Clin Nucl Med.

[23] Wo S, Matesan MC (2019). 18F-Fluciclovine uptake in celiac ganglia: a pitfall in prostate cancer PET imaging. Clin Nucl Med.

[24] Adams MC, Turkington TG, Wilson JM, Wong TZ (2010). A systematic review of the factors affecting accuracy of suv measurements. AJR Am J Roentgenol.

[25] Dadgar H, Vafaee MS, Norouzbeigi N, Jafari E, Assadi M (2021). Dual-phase 68Ga-PSMA-11 PET/CT may increase the rate of detected lesions in prostate cancer patients. Urologia.

[26] Lopci E, Lughezzani G, Castello A, Saita A, Lazzeri M (2021). Prospective evaluation of 68Ga-labeled prostate-specific membrane antigen ligand positron emission tomography/computed tomography in primary prostate cancer diagnosis. Eur Urol. Focus.

[27] Cho J, Choe JG, Pahk K, Choi S, Kwon HR, Eo JS (2017). Ratio of mediastinal lymph node SUV to primary tumor SUV in 18F-FDG PET/CT for nodal staging in non-small-cell lung cancer. Nucl Med Mol Imaging.

[28] Rowe SP, Macura KJ, Mena E, Blackford AL, Nadal R, Antonarakis ES (2016). PSMA-Based [(18)F]DCFPyL PET/CT is superior to conventional imaging for lesion detection in patients with metastatic prostate cancer. Mol Imaging Biol.

[29] Afshar-Oromieh A, Hetzheim H, Kübler W, Kratochwil C, Giesel FL, Hope TA (2016). Radiation dosimetry of 68Ga-PSMA-11 (HBED-CC) and preliminary evaluation of optimal imaging timing. Eur J Nucl Med Mol Imaging.

[30] Afshar-Oromieh A, Sattler LP, Mier W, Hadaschik BA, Haberkorn U (2017). The clinical impact of additional late PET/CT imaging with 68Ga-PSMA-11 (HBED-CC) in the diagnosis of prostate cancer. J Nucl Med.

[31] Sahlmann CO, Meller B, Bouter C, Ritter CO, Ströbel P, Lotz J (2015). Biphasic (68)Ga-PSMA-HBED-CC-PET/CT in patients with recurrent and high-risk prostate carcinoma. Eur J Nucl Med Mol Imaging.

[32] Jansen B, Yaqub M, Voortman J, Cysouw M, Windhorst A, Schuit R (2019). Simplified methods for quantification of 18F-DCFPyL uptake in patients with prostate cancer. J Nucl Med.

[33] Wondergem M, van der Zant FM, Knol RJJ, Lazarenko SV, Pruim J, de Jong IJ (2017). (18)F‐DCFPyL PET/CT in the Detection of Prostate Cancer at 60 and 120 min: detection rate, image quality, activity kinetics, and biodistribution. J Nucl Med.

[34] Schmuck S, Nordlohne S, Klot CV, Henkenberens C, Sohns JM, Christiansen H (2017). Comparison of standard and delayed imaging to improve the detection rate of [68Ga] psma I&T PET/CT in patients with biochemical recurrence or prostate-specific antigen persistence after primary therapy for prostate cancer. Eur J Nucl Med Mol Imaging.

